# Early temporal characteristics of elderly patient cognitive impairment in electronic health records

**DOI:** 10.1186/s12911-019-0858-0

**Published:** 2019-08-08

**Authors:** Somaieh Goudarzvand, Jennifer St. Sauver, Michelle M. Mielke, Paul Y. Takahashi, Yugyung Lee, Sunghwan Sohn

**Affiliations:** 1 School of Computing and Engineering, University of Missouri, Kansas City, MO USA; 2 Division of Epidemiology, Mayo Clinic, Rochester, MN USA; 3 Community Internal Medicine, Mayo Clinic, Rochester, MN USA; 4 Division of Digital Health Sciences, Mayo Clinic, Rochester, MN USA

**Keywords:** Cognitive impairment, Topic modeling, Deep learning, Activity of daily living, Early diagnosis

## Abstract

**Background:**

The aging population has led to an increase in cognitive impairment (CI) resulting in significant costs to patients, their families, and society. A research endeavor on a large cohort to better understand the frequency and severity of CI is urgent to respond to the health needs of this population. However, little is known about temporal trends of patient health functions (i.e., activity of daily living [ADL]) and how these trends are associated with the onset of CI in elderly patients. Also, the use of a rich source of clinical free text in electronic health records (EHRs) to facilitate CI research has not been well explored. The aim of this study is to characterize and better understand early signals of elderly patient CI by examining temporal trends of patient ADL and analyzing topics of patient medical conditions in clinical free text using topic models.

**Methods:**

The study cohort consists of physician-diagnosed CI patients (*n* = 1,435) and cognitively unimpaired (CU) patients (*n* = 1,435) matched by age and sex, selected from patients 65 years of age or older at the time of enrollment in the Mayo Clinic Biobank. A corpus analysis was performed to examine the basic statistics of event types and practice settings where the physician first diagnosed CI. We analyzed the distribution of ADL in three different age groups over time before the development of CI. Furthermore, we applied three different topic modeling approaches on clinical free text to examine how patients’ medical conditions change over time when they were close to CI diagnosis.

**Results:**

The trajectories of ADL deterioration became steeper in CI patients than CU patients approximately 1 to 1.5 year(s) before the actual physician diagnosis of CI. The topic modeling showed that the topic terms were mostly correlated and captured the underlying semantics relevant to CI when approaching to CI diagnosis.

**Conclusions:**

There exist notable differences in temporal trends of basic and instrumental ADL between CI and CU patients. The trajectories of certain individual ADL, such as bathing and responsibility of own medication, were closely associated with CI development. The topic terms obtained by topic modeling methods from clinical free text have a potential to show how CI patients’ conditions evolve and reveal overlooked conditions when they close to CI diagnosis.

## Background

Medical achievements have produced a population whose lifespan has increased by 30 years since the beginning of the twentieth century [1]. In 2012, there were 40.7 million people aged 65 and over in the United States (13.2% of the total population), with 38.7% reported to have one or more disabilities [2]. The aging population has also led to an increase in persons living with CI, more than 17 million people in the United States [3], causing patients, their families, and society an annual estimate of $18 billion in lost income and direct cost of care [4]. Herein, we are defining CI as either mild cognitive impairment (MCI) or dementia. In 2015 Alzheimer’s Disease International estimated that dementias affected 46.8 million individuals worldwide. They projected the number to nearly triple by 2050 reaching 131.5 million people worldwide [5]. Regarding this, the subject of MCI is paramount as it is a transitional zone between normal life in older ages and dementia. One study indicated that clinicians were not aware of CI in more than 40% of their patients [6]. The failure to diagnose cognitive complaints will delay appropriate care plans of underlying diseases and comorbid conditions, and may cause safety issues for patients and others [7, 8]. In many cases, the CI problem will worsen over time [8–10]. Thus, early diagnosis of CI can be of utmost importance and may reduce the large burden later on the medical and social care.

The impact of CI on ADL has been used as a criterion to differentiate MCI and dementia [11]. ADL is often divided into basic ADL (b-ADL), which includes activities such as personal hygiene, clothing, feeding and toileting [12] and instrumental ADL (i-ADL), which is commonly referred to as independent living abilities such as household activities, handling money, shopping, and transportation [13–15]. The i-ADL has a higher demand for cognitive function than the b-ADL and is important for living an independent life in society [16]. ADL is highly dependent on cognitive function and behavior [17]. Therefore, there should be assessments that are capable of detecting changes in ADL as soon as changes in cognition and behavior are detected [17].

In this study, we first examined basic statistics of EHR corpus relevant to CI diagnosis. Temporal trends of ADL in elderly patients (age 65 or up) mined from EHRs before they develop CI were compared between CI and CU patients. We used both structured (current visit information provided by patients) and unstructured data (clinical notes). Furthermore, we applied machine learning techniques (i.e., three topic modeling methods) on clinical notes to extract meaningful semantics (i.e., topics and terms) residing in clinical free text to examine their potential association with future CI development.

Different studies have used machine learning algorithms to differentiate between cognitively normal and MCI individuals [18, 19], to predict conversion from MCI to Alzheimer’s disease (AD) [20], and to predict the time to this conversion [19]. Researchers [21] developed two layers model in which the first layer is for a screening test to categorize a normal or abnormal group. The second layer is a close examination to classify MCI or dementia. They compared result with various machine learning approaches. Support vector machines, multi-layer perceptron and logistic regression showed high performance. Conversion from MCI to AD has also been studied using a deep learning model with MRI, neuropsychological and demographics data [22].

In another study [23], they tried to predict MCI from spontaneous spoken utterances. Classifying cognitive profiles using machine learning with fMRI data as an addition to cognitive data were explored [24]. In their work fMRI data are only used to train the classifier and classification of new data is solely based on cognitive data. Another research [25] focuses on the early diagnosis of AD with deep learning, utilizing sparse auto-encoder. They used neuroimages obtained from neuroimaging initiative database for identifying the region of brain images that are sensitive to AD progression. These previous studies tried to leverage their results by incorporating fMRI data into their models. Although it may have a positive impact on the result, not all the patients have the fMRI data so it may not be broadly applicable, compared to the application using routine EHR data in the health care population. Also, they did not try to identify new risk factors associated with the CI patients in EHRs but only rely on existing known medical conditions and fMRI data to predict the CI.

There are studies focused on predicting progression from MCI to dementia using neuropsychological data. Researches [26] considered the neuropsychological test results to examine their applicability for predicting dementia using a machine learning algorithm. They used a feature selection ensemble approach to choose the features available in the neuropsychological test as a predictor of developing AD dementia. The neuropsychological test to predict the time conversion from MCI was also investigated in [27]. In this study, MCI patients were grouped with regards to who developed to dementia (converter MCI) or remained MCI (stable MCI) during a specified time window. Then a prognostic model was developed to predict the conversion time as early as 5 years before developing to dementia.

Unlike the previous studies, we applied a machine learning approach (i.e., topic modeling) to examine topics and terms in EHR free text that can be potentially used for early detection of CI. A few studies have focused on the early diagnosis of CI [28]; however, these studies have followed the conventional approaches of assessing patients by i-ADL and b-ADL rather than utilizing machine learning algorithm and EHR free text.

## Methods

The basic EHR corpus statistics (i.e., distributions of event types and practice settings of the first CI diagnosis, numbers of clinical notes between CI and CU patients) were examined. Temporal trends of patient ADL were compared and topics in the clinical free text were analyzed over time using three machine learning models between physician-diagnosed CI and CU patient groups.

### Data

The study cohort was selected from patients 65 years of age or older at the time of enrollment in the Mayo Clinic Biobank (*n* = 22,772), where we identified physician-diagnosed CI patients (*n* = 1,435; male 55%) and CU patients (*n* = 1,435) matched by age (+/− 1 year) and sex. The physician-diagnosed CI patients were determined based on diagnosis (i.e., dementia, cognitive impairment, cognitive deficit, cognitive decline, mild cognitive impairment) under the diagnosis section in clinical notes [29].

### Corpus analysis

The basic EHR corpus statistics relevant to CI diagnosis (i.e., the distributions of event types and practice settings of the first CI diagnosis) were examined and also the number of clinical notes over time between CI and CU patients was compared.

### Analysis of activity of daily living

The ADL was collected from two sources: 1) the current visit information, which is provided and updated by the patients every 6 months when they visit the Mayo Clinic, 2) certain sections in clinical notes (i.e., instructions for continuing care, ongoing care orders, system review). The current visit information includes questionnaires to assess the ability of patients to accomplish ADL (binary assessment assessing the difficulty of ADL: yes or no) in a structured format. The clinical notes were processed by the MedTaggerIE module in MedTagger [30, 31], which is the open-source pipeline developed by Mayo Clinic for pattern-based information extraction with a capability of assertion detection (i.e., negated, possible, hypothetical, associated with a patient) and normalization, to extract ADL related concepts. These concepts were automatically mapped to the corresponding predefined ADL categories through the MedTaggerIE implementation (i.e., rule-based normalization process). We only included non-negated ADL related concepts.

Once we obtained ADL concepts, they were mapped to items in Katz’s index (b-ADL) [12] and Lawton scale (i-ADL) [13–15], which are the most commonly used tools for assessing ADL. The items of ADL used in this study for each ADL category are—1) b-ADL: bathing, dressing, transferring, toileting, and feeding; 2) i-ADL: using transportation, shopping, preparing food, housekeeping, responsibility for own medications, and handling financing. These items can be mapped to the International Classification of Functioning, Disability, and Health (ICF) [32], allowing for broad information exchange. The temporal trends of b-ADL and i-ADL between CI and CU patients were compared in every 6 months for 5 years before the first physician-diagnosed CI and the latest visit for CI and CU patients, respectively.

### Analysis of topics in clinical notes

The topics in clinical notes were investigated: 1) how topic terms evolve in CI patients each year for the past 5 years (experiment 1), and 2) how topic terms are different between CI and CU patients over the 5-year period before the development of CI (experiment 2). This step-wise time frame allows us to observe how the topics change over time, motived by the expert recommendation that people older than 65 years old should visit doctors every 6 months to determine if symptoms are staying the same, improving or growing worse [17]. We examined the topics in 1) entire clinical notes, 2) individual sections (i.e., history of present illness, diagnosis, current medication) independently, and 3) the set of sections that most likely include medical concepts of interest (i.e., chief complaint, history of present illness, system review, past medical history, physical examination, impression/report/plan, and diagnosis).

For preprocessing the topic models, we keep the most frequent 2,000 words as the vocabulary after removing stop words and stemming. We applied three different machine learning models; two conventional topic modeling methods (LDA and TKM) and one deep learning approach (KATE) as follows. The number of topics was determined based on the self-regulatory capability embedded in a TKM model.

#### Latent Dirichlet allocation (LDA)

It is a generative probabilistic model in which the document will be viewed as a mixture of various topics and each topic as a distribution of the words [33]. We set the number of topics to 20 and 10 words distribution in each topic. Other hyper parameters were set as the code implemented in [34].

#### Topic keyword model (TKM)

This method addresses the shortcoming of LDA approach (i.e., ignoring the order of words). In TKM, each word in each topic aims to show how common the word is within the topic and how common it is between other topics [35]. The other advantage of this method is that redundant topics will be removed automatically. We used the hyper parameters as explained in the paper in [35].

#### K competitive autoencoder (KATE)

An autoencoder is a neural network which can automatically learn data representations though constructing its input at the output level. Many variants of autoencoders have been proposed mainly for image data. However, KATE has been designed to overcome the weakness of traditional autoencoder which is not suitable for textual data [34]. The number of the topics in this experiment was set to 20 and 10 words distribution for each topic. Other deep learning parameters were set as discussed in the original paper [34].

## Results

We first examined basic EHR corpus statistics of the cohort. Then, we analyzed patterns of temporal trends of 1) b-ADL and i-ADL and 2) individual ADL between CI and CU patients before patients develop CI. The outcomes of three topic modeling methods (i.e., terms and topics mined from clinical notes) were analyzed and compared between the two patient groups over time, both qualitatively and quantitatively, in order to better understand patient medical conditions that may contribute more to CI development.

### Corpus statistics

**Fig. 1:**
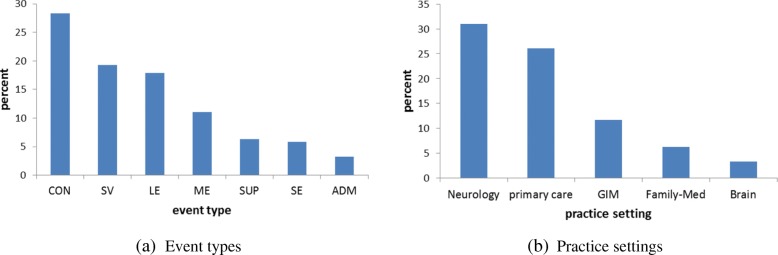
Distribution of the first CI diagnosis (CON: consult, SV: subsequent visit, LE: limited exam, ME: multi-system evaluation, SUP: supervisory, SE: specialty evaluation, ADM: admission; GIM: general internal medicine)

Figure 1 shows major event types (i.e., note types) and practice settings along with their occurrences in which a physician first diagnosed CI. The consultation was the most dominant event to diagnose CI (28%), followed by subsequent visit (19%), limited exam (18%), multi-system evaluation (11%), and supervisory (6%), which cover more than 80% of total events of CI diagnosis. For practice setting, neurology (31%) was the most dominant, followed by primary care (26%), general internal medicine (12%), family medicine (6%), and brain (3%).

Table 1 contains the statistics of clinical notes for the past 5 years of CI and CU patients before they develop CI and the latest visit date, respectively. As can be seen, CI patients consistently showed higher reading of clinical notes than CU patients and the difference was most significant in the first year before CI diagnosis.

**Table 1:** Average number of clinical notes for CI and CU patients (SD in parenthesis)

Year^a^	CI patient	CU patient
1	30.7 (41.5)	21.8 (34.0)
2	22.4 (26.4)	17.2 (24.0)
3	21.4 (25.7)	17.0 (23.5)
4	21.1 (25.7)	16.1 (20.9)
5	18.7 (21.3)	15.4 (18.7)

^a^Before CI diagnosis or latest note date for CI and CU patients, respectively

### ADL distribution

**Fig. 2:**
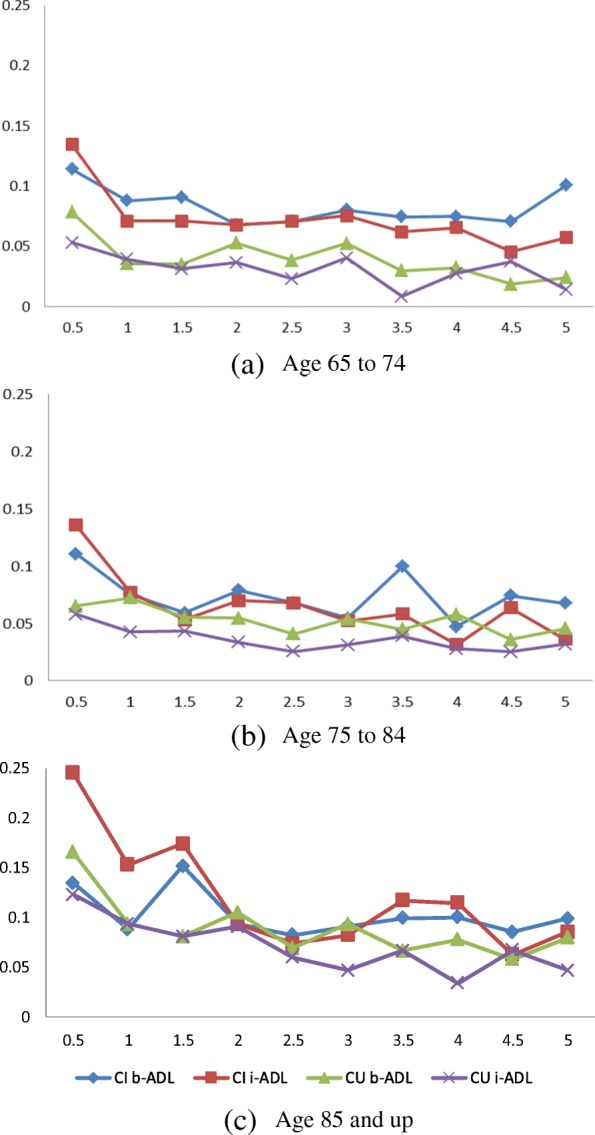
Distribution of b-ADL and i-ADL for CI and CU patient groups (x-axis is year(s) before the 1st physicain-diagnosed CI for CI patients and the latest visit for CU patients; y-axis is a ratio of patients who have a deteriorated ADL)

Figure 2 shows temporal distributions of the deteriorated b-ADL and i-ADL of CI and CU patients in three age groups (65–74, 75–84, and 85 & up). Overall, CI patients had worse b-ADL and i-ADL (i.e., a higher ratio of deteriorated ADL) than CU patients in all age groups and this trend is more significant when it is close to physician-diagnosed CI for CI patients. The deteriorated b-ADL and i-ADL between the age groups of 65–74 and 75–84 are not much different for both CI and CU patients. Interestingly, the overall CU patients’ b-ADL were worse than i-ADL, but it is opposite for CI patients—i.e., CI patients’ i-ADL became worse than b-ADL over time, mainly when it was close to 1.5 to 1 year(s) before the physician-diagnosed CI.

**Fig. 3:**
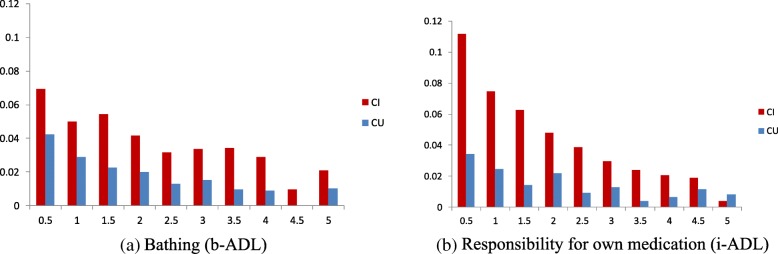
ADL distributions for CU and CI patient groups (x-axis is year(s) before the 1st physicain-diagnosed CI for CI patients and the latest clinical visit for CU patients; y-axis is a ratio of patients who have a deteriorated ADL)

We have also examined individual ADL trajectories for the entire patient cohort between CI and CU patients. Overall, CI patients had more deteriorated ADL than CU patients over time for all ADL categories. The most deteriorated ADL in 6 months prior was transferring (17% for CI and 14% for CU patients) in b-ADL and housekeeping (14% for CI and 10% for CU patients) in i-ADL. The difference between the two groups is relatively small for housekeeping and transferring, but large for bathing and responsibility for own medication (Fig. 3).

### Topic modeling

#### Qualitative analysis

We examined the topic terms extracted by three different models (i.e., LDA, TKM, and KATE) from clinical notes to compare hidden topics in CI patients before they develop CI. This approach may reveal potential patient medical conditions that lead to CI. Tables 2, 3 and 4 include topic terms generated by different topic models in different portions of clinical notes for 6 months before physician-diagnosed CI. The bold font in the topic denotes the correlated words in a given topic relevant to CI.

**Table 2:** Topic words by TKM (6 months before CI diagnosis)

Section	Word distribution for topics
All	pain **symptom** scalepati feel hpi **fatigu** numer loss **vomit** rate worst **appetit** statusth climb **headach**
Set of sections	**sleep apnea** cpap obstruct **sleepi oximetri** interfac **daytime** polysomnographi **snore**
History of present illness	**glucos sugar** metformin **pressur** blood **insulin** vitamin **diabet cholesterol losartan** interact **hydrochlorothiazide**
Medication	**sugar glucos metformin decitabin blood** pseudogout copeman **diabet losartan** fast **insulin lantu** read station **glipizide**
Diagnosis	lesion **carcinoma** cell **dermatolog melanoma ulcer cancer squamou surgeri** concern examin **nonmelanoma**

The bold font denotes the correlated words for a given topic relevant to CI

**Table 3:** Topic words by KATE (6 months before CI diagnosis)

Section	Word distribution for topics
All	mouth **hpi** releas capsul **sleep apnea** gi nasal prescript obstruct
Set of sections	medic prescript **sleep hypertens** obstruct concern **diabet apnea hyperlipidemia** acut
History of present illness	**chronic** diseas atrial **hypertens** failur **fibril** acut heart **coronari** back
Medication	drop **zocor aspirin** ophthalm day tablet low **atenolol** apr
Diagnosis	histori **sleep apnea** obstruct **hypertens** disord **hyperlipidemia** neuropathi bilater **depress**

The bold font denotes the correlated words for a given topic relevant to CI

**Table 4:** Topic words by LDA (6 months before CI diagnosis)

Section	Word distribution for topics
All	normal **distress** clear alert bilater soft **edema** sound orient tender
Set of sections	diseas histori **hypertens chronic** statu arteri atrial **hyperlipidemia coronari** diabet
History of present illness	**urinari urin** incontin bladder infect tract symptom deni histori urgenc
Medication	carbidopa levodopa hs benjamin start vitamin garlic bid knutson hydrochlorothiazide
Diagnosis	**memori hypertens** concern **hyperlipidemia** health mainten chronic **hypothyroid** complaint elev

The bold font denotes the correlated words for a given topic relevant to CI

The words in the tables are stemmed. We included one representative cluster of the topics for each section. As can be seen in the tables, the topics are distinguishable of each other, capturing a meaningful representation of the text data. For example, Table 2, *all sections* show some symptoms related to “fatigue,” which may be the potential risk of dysfunction [36]; the topic in *set of sections* is relevant to “sleep issue” that could be observed in the individuals suffering from cognitive disorder [36, 37]. The topic words in the *history of present illness* section, we can observe glucose, diabetes, insulin, and hydrochlorothiazide, which are related to diabetes disease considered as a potential risk factor of cognitive decline [38]. For the topic in the *medication* section, we observed medications to control high blood sugar [38]. The topic in the *diagnosis* section includes the terms related to cancer [39–42].

Table 3, *set of section* and *history of present illness* include hyperlipidemia that can be considered as a risk factor of CI [43], coronary artery disease and hypertension, which are relevant to cognitive decline [44, 45]. In Table 4, LDA result in similar outcomes as TKM and KATE is shown. Words like edema, distress, memory, hypertension, coronary, urinary and hyperlipidemia as the potential risk factor of cognitive dysfunction was discussed [44–47]. Carcinoma, melanoma, cancer, and squamous in the last row are the terms related to cancer [39–42].

#### Quantitative analysis

We quantified how the topic terms learned by the topic models are: 1) changed in CI patients when they approach physician-diagnosed CI, comparing year by year for the past 5 years (experiment 1), and 2) distinct between CI and CU patients for the entire past 5 years (experiment 2). We utilized aggregated term frequency in the topic terms over time.

For the first approach (experiment 1), the differences of topic term frequencies between two consecutive years prior to CI diagnosis were computed (starting from 1 year prior to the CI diagnosis), repeated for each year, for the whole 5 year period. We used 400 topic terms for each year. This may allow us to identify potential topic terms associated with CI development because we may observe more frequent topic terms that are relevant to CI when it approaches the CI diagnosis date. For the second approach (experiment 2), we also used the same approach of aggregated term-frequency differences but for the entire 5-year period. In this way, the common topic terms between CI and CU patients might be sorted out and the remaining terms are likely the ones associated with CI. The reason we used the entire 5 years was that we have not observed any significance comparing year by year.

**Fig. 4:**
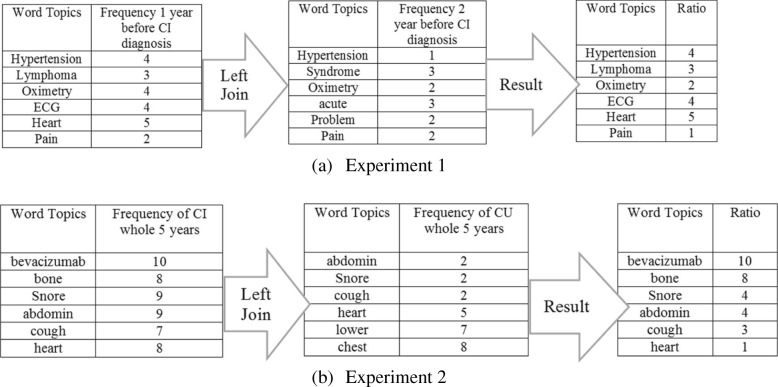
Aggregated term frequencies. The first table shows the frequency one year before CI development, middle table is the frequency two year before CI development. Last table is the result which terms repeated most

**Fig. 5:**
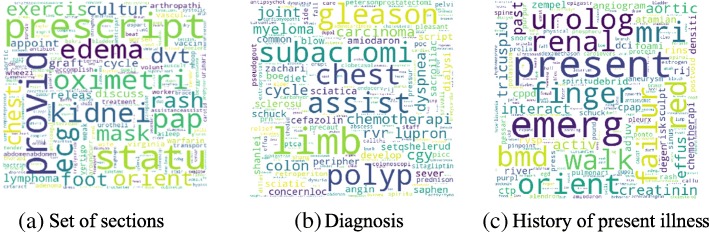
Topic terms for CI patients - TKM (Experiment 1)

**Fig. 6:**
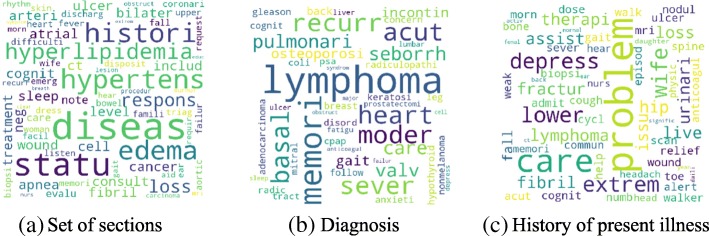
Topic terms for CI patients - KATE (Experiment 1)

**Fig. 7:**
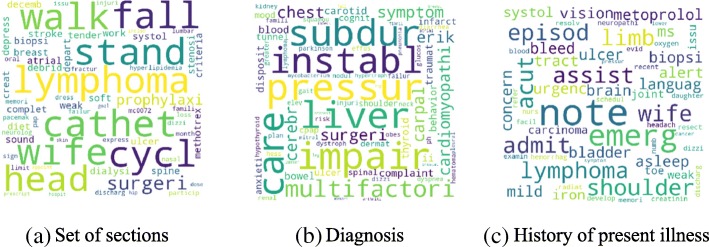
Topic terms for CI patients - LDA (Experiment 1)

**Fig. 8:**
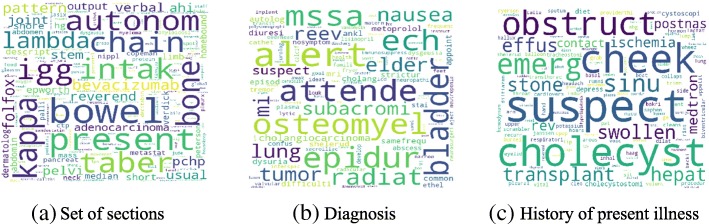
Topic terms in the TKM model (Experiment 2)

**Fig. 9:**
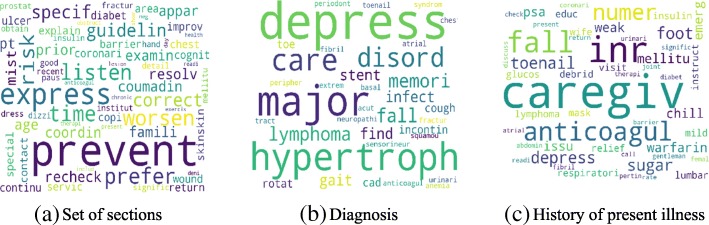
Topic terms in the KATE model (Experiment 2)

**Fig. 10:**
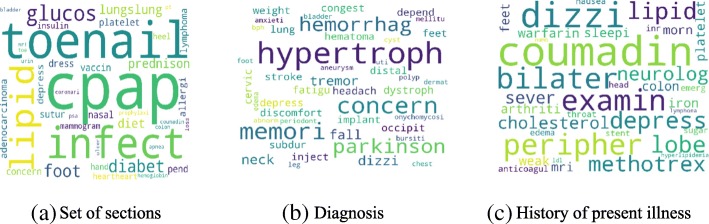
Topic terms in the LDA model (Experiment 2)

Figure 4 shows the high-level concept of our approach using aggregated term differences. The result of these approaches is visualized in Figs. 5, 6, 7, 8, 9 and 10. The larger words denote that they appear more frequently in the result of topic modeling on clinical notes compared to the previous year (experiment 1), or in the whole 5 years (experiment 2) (the corresponding individual raw data in Figs. 5, 6, 7, 8, 9 and 10 are located in Tables in Appendix). The results were compared with the recent publication to verify whether this approach generates meaningful outcomes relevant to CI.

A disease, “lymphoma” was seen in multiple results (Figs. 5a, 6b, c, 7a, b, c, 8b, 9b, c and 10a, c), which appeared in Hodgkin lymphoma patients complaining about cognitive deterioration and fatigue [48]. A researcher found that cognitive decline was more severe and frequent in Hodgkin lymphoma patients compared to the healthy population [48]. Based on recent study patients with “nocturnal hypoxia” had poor memory retention compared with healthy individuals [49]. Indeed, “oximetry” (Fig. 5a) is a device able to measure the oxygen saturated in the blood in hypoximia patients.

In another study [50], a researcher demonstrated that “global cerebral edema” is a vital risk factor for cognitive dysfunction which we see more frequently in Figs. 5a, 6a, and 10b,c. Researchers studied the association between cancer and cognitive decline in older ages [39–42]. They concluded that cancer therapy could negatively impact cognition in some patients. Regarding to this, the word “metastasi,” “squamous,” “chemotherapy,” “oxaliplatin,” and “carcinoma” can be seen in Figs. 5c, 7b, c, 8a, b, 9b, and 10a, c. It has been explored that “tinnitus patients” are more at risk of the cognitive deficit as shown in Fig. 5b, c [51]. The word “bevacizumab” in Fig. 8a is a cancer medicine that interferes with the growth of a cancer cell in the body. Indeed, it is used to treat certain types of brain cancer or kidney cancer. The relation between urinary disease and CI has been investigated in several studies (Figs. 6c and 8b) [46, 47]. The words like “depression,” “confusion,” “memory,” and “pressure,” which has been already known as the sign of CI can be seen in the Figs. 6b, 7a, b, c, 9b, c, and 10b, c.

A couple of the studies explored the relationship between CI in late life and hyperlipidemia, hypertension, and coronary (Figs. 6a, 9a, 8c, and 10c). Heavy snoring and sleep apnea in Figs. 6a, b, c and 8a have been investigated largely by researchers which shows a strong link to earlier cognitive decline [37]. An apnea/hypopnea index is an index, which is usually used to indicate the severity of sleep apnea in patients, is another extracted topic repeated 8 times more in the CI population compared with CU. CPAP is used to treat sleep-related breathing disorders including sleep apnea (Fig. 8c).

Diabetes diseases have been identified as a potential risk of cognitive dysfunction [38] and regarding that topic diabetes, glucose, and sugar [44, 45, 52] can be seen at Figs. 6c, 9c, and 10a. In [53], researchers showed that memory impairment has a particular association with the presence of left ventricular hypertrophy (Figs. 9b and 10b). Atrial Fibrillation has been studied at [54] as a risk factor of cognitive decline (Figs. 6c, 8c, and 9c). We can find the relation between “osteomyel” patients and CI at [55] as illustrated in Fig. 8b.

In [56] researcher explored that after ischemia cognitive function is disrupted (Fig. 8c). Figures 8b and 10b, c indicate the word “lung.” Some studies including researchers at [57] discussed lung diseases as a determinant of cognitive decline.

Apart from the topics and words discussed here, there are some words whose frequency was high in the years close to CI diagnosis, so they are bold and large. Some of them, for example, caregiver, care, exercise, and neuropathy may be indirectly relevant to CI. However, there are common words like boilerplate such as problem, pain, sudden, disease, status, which can appear in all diseases and need to be filtered out.

## Discussion

It is important to identify early signs of CI and thus clinicians plan accordingly and perform appropriate actions, relieving potential cost and burden. In this study, we examined basic EHR corpus statistics relevant to CI patients, and analyzed temporal trends of patient ADL over time and topics in clinical notes between CI and CU patient groups in order to characterize and better understand elderly patient’s medical conditions before they develop CI.

The consultation was the most significant event type, and the neurology was the most dominant practice setting first to diagnose CI by physicians. The consistently higher number of clinical notes for CI patients than CU patients presumably concludes that CI patients likely visit hospitals or clinics more than CU patients. Temporal trends of individual ADL and the groups of ADL (i.e., b-ADL and i-ADL) have been examined over time back in 5 years before the first physician-diagnosed CI and the latest visit for CU patients, respectively. It was observed that the trajectories of ADL deterioration became steeper in CI patients than CU patients approximately 1 to 1.5 year(s) before the actual physician diagnosis of CI. More notably, the deterioration of i-ADL was worse than that of b-ADL in CI patients during this period, which was not in the case in CU patients. Considering a significant delay in CI diagnosis and a missing opportunity for appropriate plans in the current practice [4, 5], this observation may be beneficial to promote early detection of CI. The trajectories of bathing (b-ADL) and responsibility for own medication (i-ADL) deteriorated much more rapidly in CI patients than CU patients over time. These measures might also be a potential surrogate symptom to facilitate early CI diagnosis.

The result of this study suggests that using topic modeling can benefit to discover meaningful and hidden topics and terms of the clinical notes. The result was promising as we discussed in the qualitative and quantitative analysis. We observed that the words in the topic were mostly correlated and captured the underlying semantics. The model was able to extract the words relevant to CI; the words like hypertension, depress, and memory which are a potential indication associated with CI. We were also able to come up with other potential factors that may be relevant to CI according to the recent publications.

Overall, the recent models TKM and KATE were better at capturing the semantically meaningful representation of the data compared to LDA. Further, KATE model generated more words related to CI which falls in memory, depression, hypertension, dizziness, and confusion category than TKM model. We validated the results of the topic modeling based on aggregated term frequencies. The results were visualized to show the hidden potential topics that may contribute to developing CI. These results were validated by recent publications and showed promising outcomes. However, some common topic words, not relevant to CI but may appear in any diseases, were also captured. A further post-process would be required to filter out them.

Generally, CI is diagnosed by health professionals through asking questions to patients to assess memory, concentration, and understanding. However, it is not routinely performed in many healthcare institutions, causing a delay in timely CI diagnosis. Considering this fact, our study of the use of EHR free text to analyze early signals of CI would be a potential alternative to automate or support CI assessment and thus to facilitate a routine practice to detect CI in advance.

The limitations of this study include the use of physician-diagnosed CI, which does not differentiate the severity of CI, instead of full assessment or test due to its unavailability. However, our study is still useful since the focus of this study is to explore the use of EHR documentation to promote early detection of CI, considering the significant delay in CI diagnosis by clinicians in the current health care practice. Another limitation would be a potential imbalanced distribution of clinical notes for certain illnesses (e.g., cancer patients are seen more than others and have more clinical notes). This may affect the result of topic modeling; however, we examined a broad range of topics and demonstrated good potential applicability.

## Conclusion

There exist notable differences in temporal trends of b-ADL and i-ADL between CI and CU patients, approximately 1 to 1.5 year(s) earlier than actual physician-diagnosis CI—i.e., the steeper slope of overall ADL deterioration and worse i-ADL than b-ADL in CI patients during this period. The trajectories of certain individual ADL (bathing and responsibility of own medication) were closely associated with the CI development. The topics and terms over time obtained by topic modeling methods from clinical free text have the potential to show how CI patient’s conditions evolve and reveal overlooked conditions when they close to CI diagnosis. These observations may promote early detection of CI and thus expedite appropriate care of underlying diseases and comorbid conditions. In the future, we plan to use neuroimaging and assessment data to identify the more granular classification of cognitive function and develop a prediction model leveraging our observations to detect patients in high risk of different stages of CI and identify associated longitudinal risk factors.

## Appendix

Tables 5, 6, 7 contain sample raw data in experiment 1, corresponding to Figs. 5, 6 and 7. It illustrates how many times of a given term appeared more in clinical notes of CI patients than the previous year.

**Table 5:** Raw data used in Fig. 5 TKM Experiment 1

Set of sections		Diagnosis		History of present illness	
Term	Rate of increase	Term	Rate of increase	Term	Rate of increase
provid	4	limb	4	emerg	4
prescript	4	chest	3	present	4
statu	4	chemotherapi	3	walk	3
kidnei	3	cgy	3	renal	3
oximetri	3	dyspnea	3	bmd	3
pap	3	myeloma	3	chemotherapi	2
ecg	3	carcinoma	3	cpap	2
edema	3	cefazolin	2	snore	2
lymphoma	3	pressure	2	pulmonari	2
asleep	2	bevacizumab	2	diabet	2
urinary	2	hyperlipidemia	2	epworth	2
hypertrophy	2	neuropathi	2	bladder	2
hypersensitiviti	2	metastas	2	thyroid	2
Insulin	2	melanoma	2	ischemia	2
lung	2	Sugar	2	thyroid	2
memory	2	Warfarin	2	insulin	2
numb	2	lung	2	potassium	2
symptom	1.5	ldl	2	fibril	1.5
cough	1.5	Sleep	1.5	assist	1.5
atrial	1.5	Medic	1.5	symptom	1.5

**Table 6:** Raw data used in Fig. 6 KATE Experiment 1

Set of sections		Diagnosis		History of present illness	
Term	Rate of increase	Term	Rate of increase	Term	Rate of increase
disease	7	lymphoma	6	problem	7
Status	6	memori	5	care	6
hypertens	5	acut	4	lower	4
edema	5	heart	4	extrem	4
hyperlipidemia	5	pulmonari	3	wife	4
respons	4	care	3	depress	4
loss	4	anxiety	2	hip	3
sleep	3	adenocarcinoma	2	lymphoma	3
bilater	3	cpap	2	assist	3
cancer	3	hypothyroid	2	loss	3
arteri	3	gleason	2	fibril	3
apnea	3	psa	2	urinary	3
fibril	3	melanoma	2	cough	2
note	2.5	Fatigue	2	memori	2
care	2.33	depress	1.8	hear	2
evaluation	2.33	sleep	1.8	headach	2
Coronary	2	anticoagul	1.67	memori	2
nurs	1.67	obstruct	1.6	back	1.67
symptom	1.62	major	1.5	ct	1.5
breath	1.5	syndrom	1.5	ear	1.5

**Table 7:** Raw data used in Fig. 7 LDA Experiment 1

Set of sections		Diagnosis		History of present illness	
Term	Rate of increase	Term	Rate of increase	Term	Rate of increase
lymphoma	3	pressur	3	note	5
walk	3	impair	3	emerg	4
cycl	3	subdur	3	limb	3
fall	3	liver	3	lymphoma	3
head	3	chest	2	asleep	2
surgeri	3	surgeri	2	neuropathi	2
prophylaxi	3	bowel	2	memori	2
atrial	2	traumat	2	dizzi	2
systol	2	thyroid	2	numb	2
depress	2	anxieti	2	issue	2
weak	2	mood	2	joint	2
dialysi	2	spinal	2	toe	2
dizzi	2	glucose	2	blood	2
dress	2	hypothyroid	2	hemorrhag	2
pap	2	cpap	2	examin	2
express	2	squamou	2	headach	2
memori	2	1.5	1.5	pressur	1.5
dose	1.67	atrial	1.5	lower	1.5
skin	1.5	memori	1.5	symptom	1.5
prescript	1.5	fibril	1.5	cancer	1.5

Tables 8, 9, 10 contains sample raw data in experiment 2, corresponding to Figs. 8, 9 and 10. It illustrates how many times a given term appeared more in clinical notes of CI patients than CU patients for the past 5 years.

**Table 8:** Raw data used in Fig. 8 TKM Experiment 2

Set of sections		Diagnosis		History of present illness	
Term	Rate of increase	Term	Rate of increase	Term	Rate of increase
bowel	12	alert	7	suspect	6
present	11	mssa	6	cholecyst	6
autonom	10	epidur	6	obstruct	5
igg	10	ech	6	emerg	5
chain	10	bladder	6	ischemia	4
bevacizumab	10	osteomyel	6	depress	4
folfox	10	lung	5	diet	4
ahi	9	nausea	5	swollen	3.5
epworth	9	tumor	5	cpap	3
snore	9	confus	4	potassium	3
abdomin	8	difficulti	4	lung	3
myeloma	7	plasma	3.5	bladder	2.5
tinnitu	6	dysgeusia	3.5	confus	2.33
housekeep	6	anxieti	3	plasma	2.33
Sleep	4.5	cefazolin	3	concern	2.25
Cough	4.5	canal	3	immune	2
dyspnea	4	bursa	2.5	metastas	2
urinari	3.75	abdomin	2.33	surgery	2
hypersensitivity	3	descript	2.25	hyperlipidemia	2
melanoma	2.67	lymphoma	2	thyroid	2

**Table 9:** Raw data used in Fig. 9 KATE Experiment 2

Set of sections		Diagnosis		History of present illness	
Term	Rate of increase	Term	Rate of increase	Term	Rate of increase
prevent	12	depress	12	caregiv	14
listen	6	hypertroph	10	inr	11
risk	6	care	8	fall	9
correct	5	memori	5	anticoagul	8
specif	5	lymphoma	5	depress	5
examin	4.5	infect	4.5	toenail	5
coumadin	4	gait	4	sugar	5
coordin	3.5	stent	4	psa	4
dizzi	3	cough	3	mellitu	4
dress	3	cad	3	issu	4
mellitu	3	toe	3	warfarin	4
prostat	3	anemia	2.5	lymphoma	3
chest	3	sensorineur	2.43	glucos	3
diabet	3	chest	2.2	insulin	3
servic	3	squamou	1.75	coronari	2.33
insulin	3	fibril	1.74	abdomin	2.25
prostat	3	urinari	1.72	diabet	2.22
coronari	2.67	syndrom	1.62	fibril	2.2
anticoagul	1.83	anticoagul	1.55	atrial	2
lesion	1.5	atrial	1.52	urinari	1.67

**Table 10:** Raw data used in Fig. 10 LDA Experiment 2

Set of sections		Diagnosis		History of present illness	
Term	Rate of increase	Term	Rate of increase	Term	Rate of increase
cpap	7	hypertroph	9	reveal	9
toenail	7	memori	7	coumadin	7
infect	6	concern	7	dizzi	6
glucos	5	hemorrhag	6	bilater	5
lipid	5	parkinson	6	depress	4.5
diabet	4	dizzi	4	bleed	4
lung	4	neck	4	neurolog	4
concern	3.33	depress	3.2	platelet	3
allergi	3	headach	3	sleepi	3
lymphoma	3	fatigu	3	warfarin	2.67
depress	3	weight	3	inr	2.5
adenocarcinoma	3	lung	3	head	2.33
insulin	3	dystroph	3	hyperlipidemia	2
toe	2.33	stroke	3	numb	2
psa	2.33	aneurysm	2.5	edema	2
hemoglobin	2	polyp	2.33	sugar	2
bladder	2	anxieti	2.33	anticoagul	2
prophylaxi	2	edema	2.25	dl	1.75
coronari	1.83	bladder	1.83	ldl	1.67
apnea	1.67	mellitu	1.5	lymphoma	1.5

In the following tables, “Rate of increase” denotes number of times for a given term appeared more for the first year before CI diagnosis compared to the previous year (Tables 5, 6, 7) or for the past 5 years (Tables 8, 9, 10).

## Abbreviations

AD: Alzheimer’s disease
ADL: Activity of daily living
CI: Cognitive impairment
CU: Cognitively unimpaired
EHR: Electronic health record
fMRI: functional magnetic resonance imaging
ICF: International classification of functioning, disability and health
KATE: K competitive autoencoder for text
LDA: Latent Dirichlet allocation
MCI: Mild cognitive impairment
TKM: Topic keyword model
